# A Latent Profile Analysis of the Consensual and Non-Consensual Sexting Experiences among Canadian Adolescents

**DOI:** 10.1177/0044118X231202814

**Published:** 2023-09-30

**Authors:** Brett Holfeld, Faye Mishna, Wendy Craig, Samar Zuberi

**Affiliations:** 1 Memorial University of Newfoundland, Corner Brook, Canada; 2 University of Toronto, ON, Canada; 3 Queen’s University, Kingston, ON, Canada

**Keywords:** sexting, adolescence, latent profile analysis, cyber bullying, self-regulation

## Abstract

Different patterns of sexting behaviors were examined to provide a more nuanced understanding of the context in which sexting occurs among adolescents. Participants were 1,000 Canadian adolescents (50.2% girls) between 12 and 18 years (*M*_age_ = 15.21, *SD* = 2.00) who completed measures of sexting, cyber bullying and victimization, problematic social media use, self-regulation, and demographics. Contrary to our hypotheses, three latent profiles of sexting represented the frequency of sexting rather than whether the sexting was consensual versus non-consensual or with a partner versus non-partner. Participants in the *moderate* and *high sexting* profiles representing one fifth of youth, reported less self-regulation, experienced more cyber victimization, and engaged in more cyber bullying and problematic social media use than those in the *no/low sexting* profile. Our findings support the normalcy approach to education, which considers some sexting among healthy developmental behaviors.

Sexting, the digital exchange of sexually explicit texts, photos, or videos (Van Ouytsel et al., 2017), has “become a part of teenagers’ everyday lives” (Van Ouytsel et al., 2019, p. 346). Likely due to a lack of standardized definition, the prevalence of sexting varies across studies (Barrense-Dias et al., 2017). The research has been guided by two contrasting frameworks. A deviancy perspective proposes that youth sexting is a risky and harmful behavior (Van Ouytsel et al., 2018) that is associated with other risky behaviors such as substance use (Frankel et al., 2018) and unprotected sex (Rice et al., 2018), as well as with emotional, interpersonal, and mental health issues (Rhyner et al., 2017). The normalcy perspective, in contrast, contends that consensual sexting is a normative risk-taking behavior (Cooper et al., 2016; Döring, 2014) and a healthy expression of adolescent sexuality (Maes & Vandenbosch, 2022; Naezer & van Oosterhout, 2021). This perspective recognizes non-consensual sexting as problematic and a type of sexual harassment (Henry et al., 2018; Jørgensen et al., 2019). A categorical approach labeling sexting as deviant or normative may be too simplistic. It is important to consider the conditions associated with the sexting, including whether the sending or receiving of sexts is consensual or non-consensual and who is engaged (i.e., with a partner or non-partner) (Krieger, 2017). The current study extends previous research by taking a person-centered approach to examine individual differences in adolescents’ sexting behaviors (sending, receiving, and distributing; consensual and non-consensual; partner and non-partner). This analysis referred to as latent profile analysis is an advanced statistical technique used to identify groups or clusters of people who share similar characteristics or have similar experiences. In this study, the analysis is particularly useful to understand the continuum of risk associated with adolescent sexting.

## Consensual Versus Non-Consensual Sexting

Consensual versus non-consensual involvement is a critical aspect of sexting behavior (Choi et al., 2016; Mori et al., 2020). Consensual sexting is often used as a safe experimental phase of sexual engagement (Burkett, 2015) and a way to demonstrate trust (Van Ouytsel et al., 2017) and sustain intimacy in long-term or long-distance relationships (Walker et al., 2013). Youth describe consensual sexting as a way to flirt or show affection at the beginning of a relationship (Van Ouytsel et al., 2017) or to maintain an established relationship (Van Ouytsel et al., 2020), to feel excitement or risk (Walker et al., 2013), to initiate reciprocity (i.e., receive a sext in return) (J. Ringrose et al., 2021), and to “joke around” or have fun (Lippman & Campbell, 2014). Consensual sexting may thus be a low-risk type of behavior.

Non-consensual sexting includes the sending and receiving of unsolicited sexts (e.g., dick pics) and the non-consensual sharing of sexts to a third party without the consent of the sext’s subject(s) (Mishna et al., 2023; B. Ringrose et al., 2022). Social pressure, which appears to be a gendered phenomenon, complicates the divide between consensual and non-consensual sexting. Young women report being commonly barraged by persistent requests for sexts (Mishna et al., 2023; Thomas, 2018). Similarly, female adolescents predominantly report experiencing pressure from their male peers to engage in sexting in the form of coercion, threat, or bribery (Maes & Vandenbosch, 2022; Sparks, 2022). Such requests exert sexual pressure on young women, described as “working a yes out” (Sanday, 2007), a phenomenon observed in fraternity culture. Such pressure leads to girls engaging in what has been described as “unwanted but consensual sexting” (Drouin & Tobin, 2014, p. 412). The pressure to engage in sexting may represent a level of risk, which can contribute to harm. Similarly, risk in sexting occurs when boys are pressured to collect sexts from girls and to non-consensually distribute these images among their male peers (J. Ringrose et al., 2021). The phenomena of boys sending unwanted dick pics or requesting sexts from girls can be understood from the viewpoint of sexual socialization, whereby sharing images of girls becomes normative and a type of social currency providing an indication that the boys are considered “man enough” (Roberts et al., 2021). Responding to this pressure to coerce girls to send images can thus represent the boys causing harm to the girls.

A study examining sexting behaviors among 543 Belgian adolescents aged 12 to 18 during a COVID-19 lockdown period found that youth who experienced the most pressure from a partner were more likely to engage in explicit sexting practices (Maes & Vandenbosch, 2022). To understand how sexting is associated with risk for other problematic behaviors, it is necessary to consider the frequency of sending, receiving, and distributing sexts and whether this behavior is consensual. Distinguishing consensual and non-consensual sexting promotes understanding and addresses youth’s experiences of online harm (Krieger, 2017; Lippman & Campbell, 2014).

## Sexting With a Partner Versus a Non-Partner

Another dimension that warrants attention is with whom youth are sexting. Sexting with non-partners may occur to gain attention from others, to experiment with sexuality when not yet sexually active, or to show interest in a potential partner during the flirtation stage (Lenhart, 2009). There may be greater harm in sexting with non-partners (Döring, 2014; Van Ouytsel et al., 2018), as sexting outside a stable relationship can be pursued for excitement and thrill and increases the likelihood of misuse and exploitation in online spaces (Bianchi et al., 2021). In contrast, consensual sexting in partnered relationships may be considered less harmful, as sexting with a partner can be considered a low-risk activity that is mutually agreed upon in the context of the relationship (Van Ouytsel et al., 2018). For example, some research with adolescents has found that sexting with a partner is associated with increased sexual and relationship satisfaction (e.g., Parker et al., 2013), as well as feelings of pleasure, relief, and empowerment (Razi et al., 2020).

## Harms Associated With Sexting

A systematic review of the literature on sexting among youth revealed a continuum of outcomes, with abusive or traumatic outcomes at one end, stigmatizing and personally harmful outcomes in the middle and positive outcomes at the other end (Doyle et al., 2021). Such diverse outcomes associated with sexting confirm the need to identify the conditions that heighten and minimize the risks associated with sexting (Drouin & Tobin, 2014). In a review of 88 studies, links were found between being female and sexting and an increased risk of cyber bullying (e.g., being bullied through technology) (Cooper et al., 2016). Gendered and sexualized cyber bullying is shaped by the normalization of gender and sexual stereotypes, with similar processes found in youth sexting (Mishna et al., 2020). Sexting that involves pressure, non-consensual sharing, and bullying and harassment, tends to reflect rigid gender and sexual stereotyping (Dobson, 2019). Such sexting “intersects with teen sexual risk-taking in general, relationship violence, bullying, and off-line sexual exploitation” (Finkelhor et al., 2020, p. 6). In a sample of early adolescents aged 11 to 14, the findings show that being asked to send sexts and receiving unsolicited or unwanted sexts was associated with psychosocial issues, including emotion dysregulation (e.g., difficulty in managing emotions appropriate to the situation) (Lu et al., 2021). In contrast, in the same study, there was no association between consensual sexting and psychosocial health (Lu et al., 2021). Research has demonstrated an association between forwarding or distributing sexts and increased usage of social media applications (Boer et al., 2021; Molla-Esparza et al., 2021).

To date, research has primarily examined sexting using a variable-centered approach. In the current research, we take a person-centered approach to test whether there were latent profiles of sexting behaviors (i.e., sending/receiving/distributing, consensual/non-consensual, and partner/non-partner) and if some profiles were more likely to represent a higher risk for problematic behaviors. Specifically, we wanted to test whether all sexting was associated with problematic behaviors (the deviancy perspective), or rather, identify what types of sexting experiences were associated with what types of problematic behaviors (the normalcy perspective). First, it was hypothesized that the profiles would be differentiated by involvement versus non-involvement in sexting; consensual versus non-consensual sexting; and partner versus non-partner. Second, it was expected that profiles with greater frequency of sexting involvement, non-consensual sexting, and non-partner sexting would report higher levels of pressure to sext, greater involvement in cyber bullying (e.g., bullying others and being bullied through technology respectively), more problematic social media use (e.g., greater difficulty controlling time spent on and behaviors associated with social media use), and less ability to self-regulate.

## Method

### Participants and Procedure

A nationally representative sample of participants from every province and territory across Canada were recruited by a Canadian research firm Environics between September and October 2020. Environics employed various strategies (e.g., targeted advertisements) to recruit parents of youth under 18 years of age and participants who were 18 years old. Environics asked for participant names, parent/guardian names, postal codes, and email addresses for those who were 18 years old. Within Environics’ participant pool, a sample of parents and adolescents were randomly selected based on the desired age characteristics of the current study. Approximately 67% of parents and adolescents contacted agreed to participate. Parents and adolescents gave consent through social media (e.g., Facebook, Instagram, LinkedIn) and via telephone (for individuals 18 years of age). Participants received a 20 gift-card for their participation. All procedures were approved by the relevant Research Ethics Boards.

Following the consent procedure, participants completed the survey online. Participants were 1,000 adolescents ranging in age from 12 to 18 (*M*_age_ = 15.21, *SD* = 2.00) with 50.2% reporting their gender identity as a girl, 48.6% as a boy, and 1.2% as non-binary. Most youth identified their race/ethnic background as White/European (72.4%) followed by Chinese (6.9%), South Asian (6.1%), Black (5.8%), Filipino (3.1%), Southeast Asian (2.6%), First Nations (2.3%), Latin American (2.0%), Other (2.0%), Arab (1.8%), Metis (1.7%), Japanese (0.6%), Korean (0.5%), West Asian (0.5%), and Inuit (0.1%). Youth could pick multiple options and were classified as racialized or not depending on having reported an exclusively White/European background or not (72.4 to 27.6%).

### Measures

#### Sexting

First, participants were presented with a definition of “sexts” (e.g., sexually explicit written content, pictures, and/or videos of oneself transmitted via technology). Next, participants were asked about their experiences sending (4-items), receiving (4-items), and distributing (3-items) sexts since March 2020. To differentiate sexting experiences, participants were asked whether the sext(s) were consensual or non-consensual, and with a partner (e.g., romantic, sexual, or dating) or non-partner (e.g., friend, acquaintance, or stranger). Each item was rated on a 4-point Likert scale (1 = *never*, 2 = *from 1 to 3 times*, 3 = *from 4 to 10 times*, 4 = *more than 10 times*). Participants were also asked about the pressure to be involved in sexting since March 2020. Specifically, participants indicated how much pressure they felt to send and receive sexts from a partner, and how much pressure they felt to send and receive sexts from someone who is not their partner. For all 4-items, participants rated the experience on a 5-point Likert scale ranging from 1 (*not at all pressured*) to 5 (*very pressured*).

#### Cyber Bullying and Victimization

Consistent with previous research from the Health Behavior Survey of School-aged Children (Cosma et al., 2020), participants’ experiences with cyber bullying and victimization were assessed with single items respectively. For example, participants were asked, “how often have you bullied other youth electronically in the past four weeks?” and “how often have you been bullied electronically by other youth in the past four weeks?” on a 4-point Likert scale ranging from 1 (*never*) to 4 (*more than 4 times*).

#### Problematic Social Media Use

Participants’ experiences using social media in the past year were assessed using the 9-item Social Media Disorder Scale (Regina et al., 2016). Specifically, participants were asked their preoccupation with social media (e.g., “regularly found you that you can’t think of anything else but the moment you will be able to use social media again?”), tolerance of social media (e.g., “regularly felt dissatisfied because you wanted to spend more time on social media?”), withdrawal for social media (e.g., “often felt bad when you could not use social media?”), persistence with social media (e.g., “tried to spend less time on social media, but failed?”), displacement with social media (e.g., “regularly neglected other activities because you wanted to use social media?”), problems associated with social media use (e.g., “regularly had arguments with others because of your social media use?”), deception regarding social media use (e.g., “regularly lied to your parents or friends about the amount of time you spend on social media?”), and conflict associated with social media use (e.g., “had serious conflict with your parents, brother(s), or sister(s) because of your social media use?”). Participants responded yes or no to each item. Responses were summed with higher scores representing more problematic social media use (e.g., greater difficulty controlling time spent on and behaviors associated with social media use).

#### Self-Regulation

Participants’ self-regulation skills were captured using the 13-item Short-Term Adolescent Self-Regulatory Inventory (ASRI; Moilanen, 2007). The ASRI is a multi-faceted measure that considers adolescents’ ability to manage how they think, feel, focus their attention, and behave. Previous research has supported the reliability and validity of the ASRI (Bergen-Cico et al., 2015). Each item (e.g., “When I’m sad, I can usually start doing something that will make me feel better”) was rated on a 5-point Likert scale ranging from 1 (*not at all true for me*) to 5 (*really true for me*). After reverse coding, items were averaged with higher scores indicating greater self-regulation skills (α = .79).

#### Demographics and Covariates

Participants were asked to self-report their age, gender, and race/ethnicity. Participants were also asked to indicate any change in daily sexting experiences since March 2020 (e.g., “To what extent has the amount of time you spent ‘sexting’ each day changed?”) on a 5-point Likert scale ranging from 1 (*a lot less*) to 5 (*a lot more*).

### Plan for Data Analysis

An iterative approach was used to fit a series of latent profiles for the eleven continuous sexting indicator variables in Mplus *version 8.6* (Muthen & Muthen, 1998/2017). Model fit statistics, interpretability, and parsimony were used to determine the optimal number of latent profiles. Model fit indices such as the Akaike Information Criterion (AIC), Bayesian Information Criterion (BIC), and Sample-Size Adjusted Bayesian Information Criterion (a-BIC) were used with smaller values indicating better fit to the data (Nylund et al., 2007). The Vuong-Lo-Mendell-Rubin test (VLMR; Lo et al., 2001) and the Lo-Mendell Rubin adjusted likelihood ration test (LMRT) were used to compare the relative model fit of latent profiles. A significant *p* value for the VLMR or LMRT indicates that a solution with k-profiles is an improvement in model fit versus a solution with k-1 profiles whereas a non-significant *p* value indicates that the model with k-1 profiles is a more optimal solution. Entropy scores and profile counts were used to evaluate the overall quality of classification. Entropy values range from 0 to 1 with values closer to 1 indicating greater accuracy in the percentage of participants classified (Nylund-Gibson & Choi, 2018). Solutions that included small profile counts (e.g., under 10% of the sample) were rejected.

After the optimal number of latent profiles were identified, demographic (e.g., age and gender) and control variables (e.g., change in sexting experiences since March 2020) were added to examine associations with latent profile membership. Next, the latent profiles were used to predict distal outcomes (e.g., self-regulation, cyber victimization, cyber bullying, and problematic social media use) beyond the effects of the covariates. The manual BCH method (Asparouhov & Muthen, 2021) was used in Mplus to carry out these analyses. In the first step, the covariates and distal outcomes were added to the auxiliary command to be saved as BCH weights. In the second step, the auxiliary model was estimated from the latent profiles using the BCH weights. A series of pairwise comparisons were employed using the MODEL CONSTRAINT command to test for differences in distal outcomes across latent profiles beyond the effect of the covariates. A more stringent *p* value of .01 was used to account for the testing of multiple contrasts.

## Results

Means and standard deviations for the latent sexting indicator variables are shown in Table 1. Model fit indices are presented in Table 2 for solutions with one to five latent profiles. Values for AIC, BIC, and a-BIC decreased across latent profiles with the lowest values found for the five-profile solution. Examination of relative model fit indices (e.g., VLMR, LMRT), however, suggests that the three-profile solution was optimal. Specifically, tests of VLMR and LMRT were not significant for the four-profile solution indicating that the solution with k-1 profiles or the three-profile solution was more appropriate. Further, low profile counts were found across the four and five profile solutions. Taken together, the three-profile solution which appeared as the most interpretable was retained for subsequent analyses.

**Table 1.** Descriptive Statistics for Latent Sexting Profile Indicators.

Latent sexting indicator	*M*	*SD*	Range
Sent consensual sexts to my partner	2.08	1.13	1–4
Sent unsolicited sexts to my partner	1.58	0.92	1–4
Received consensual sexts from my partner	1.89	1.09	1–4
Received unsolicited sexts from my partner	1.56	0.93	1–4
Sent consensual sexts to a non-partner	1.37	0.82	1–4
Sent unsolicited sexts to a non-partner	1.27	0.67	1–4
Received consensual sexts from a non-partner	1.34	0.74	1–4
Received unsolicited sexts from a non-partner	1.38	0.78	1–4
Received a forwarded sexual image and/or video from someone who was not the original sender	1.34	0.74	1–4
Forwarded a sexual image and/or video of another individual without their permission	1.26	0.68	1–4
Had a sexual image and/or video of myself forwarded without permission	1.25	0.69	1–4

**Table 2.** Model Fit Indices for Latent Profiles of Sexting Experiences.

Number of profiles	Akaike (AIC)	Bayesian (BIC)	SSA BIC (a-BIC)	Profile counts	Entropy	VLMR test *p* value	LMRT test *p* value
1	19070.12	19177.53	19107.66	975	NA	NA	
2	12026.80	12192.80	12084.82	840, 135	.99	<.001	<.001
**3**	**10309.06**	**10533.66**	**10387.56**	**794, 100, 81**	**.99**	**.015**	**.017**
4	9601.29	9884.48	9700.27	773, 66, 60, 76	.99	.528	.531
5	9068.52	9410.29	9187.97	19, 67, 760, 73, 56	.99	.216	.218

*Note.* Bolded lines represent the best fitting model. SSA = sample-size adjusted; VLMR = Vuong-Lo-Mendell-Rubin test; LMRT = Lo-Mendell Rubin adjusted likelihood ratio test.

**Figure 1.**
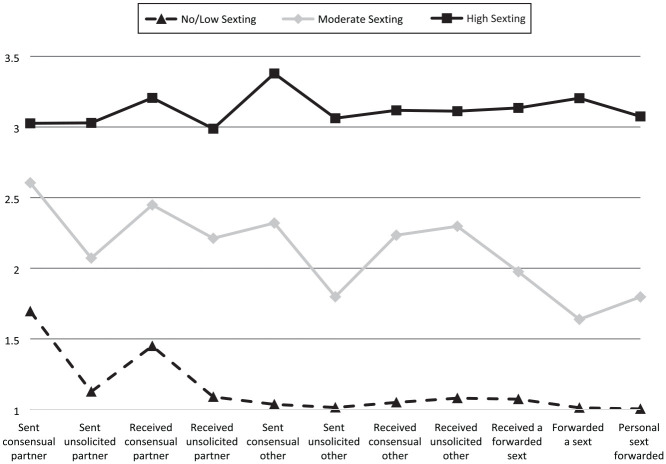
Latent sexting profiles.

Item-conditional response probabilities were used to depict the overall pattern of involvement in sexting by latent profile (see Figure 1). Categories for the latent profiles were created based on the frequency of sexting experiences. For example, the *no/low sexting* profile (*n* = 794; 81.4%) included adolescents who reported few, if any, experiences of sexting. The *moderate sexting* profile (*n* = 100; 10.3%) included adolescents who reported some experience sending and receiving consensual and non-consensual sexts to a partner and non-partner as well as a lower frequency of distributing sexts. The *high sexting* profile (*n* = 81; 8.3%) included adolescents who reported heavy involvement in sending, receiving, and distributing sexts (e.g., item averages of 3 or more out of 4) to a partner and non-partner.

Next, differences in latent profile membership across the covariates of gender, age, and change in sexting were examined. No significant differences in gender or age were found across the three latent profiles. Participants in the *moderate sexting* profile compared to the *no/low sexting* profile were more likely to report an increased change in sexting since March 2020 (*b* = 0.43, *p* = .011).

### Latent Sexting Profiles Predicting Distal Outcomes

Latent profile membership was used to predict distal outcomes controlling for the effect of the covariates (see Table 3). Significant mean level differences were found across the latent profiles for each of the four distal outcomes. The *no/low sexting* profile reported greater levels of self-regulation (*M* = 4.76, *SE* = 0.12) than the *moderate sexting* (*M* = 4.48, *SE* = 0.14) and *high sexting* profiles (*M* = 4.54, *SE* = 0.13). Experiences with technology were also related to latent profile membership, with the *no/low sexting* profile reporting less cyber victimization (*M* = 1.81, *SE* = 0.04) than the *moderate sexting* (*M* = 2.70, *SE* = 0.15) and *high sexting* profiles (*M* = 3.48, *SE* = 0.18). Similarly, the *no/low sexting* profile reported less cyber bullying (*M* = 1.96, *SE* = 0.07) than the *moderate sexting* (*M* = 2.93, *SE* = 0.18) and *high sexting* profiles (*M* = 4.30, *SE* = 0.30). The *high sexting* profile reported more cyber victimization and cyber bullying than the *moderate sexting* profile. Finally, the *no/low sexting* profile reported less problematic social media use (*M* = 0.92, *SE* = 0.03) than the *moderate sexting* (*M* = 1.58, *SE* = 0.10) and the *high sexting* profiles (*M* = 1.86, *SE* = 0.12).

**Table 3.** Standardized Mean and Standard Errors of Distal Outcomes by Latent Sexting Profile.

	No/low sexting (*n* = 794)	Moderate sexting (*n* = 100)	High sexting (*n* = 81)
Self-regulation	4.76 (.12)^a^	4.48 (.14)^a^	4.54 (.13)^a^
Cyber victimization	1.81 (.04)^a^	2.70 (.15)^a,b^	3.48 (.18)^a,b^
Cyber bullying	1.96 (.07)^a^	2.93 (.18)^a,b^	4.30 (.30)^a,b^
Problematic social media use	0.92 (.03)^a^	1.58 (.10)^a^	1.86 (.12)^a^

*Note*. Row entries with different letter superscripts denote profiles that are significantly different from each other at *p* < .01.

### Differences in Consensual and Non-Consensual Sexting Items Across Latent Profiles

To verify whether differences between the sending and receiving of consensual and non-consensual sexting items were statistically significant across latent profiles, a series of one-way ANOVA’s were used. Tests of homogeneity of variance using Levene’s *F* test were significant, indicating unequal variances. Thus, a corrected test, the Welch test was used for each one-way ANOVA (Field, 2013). There was a significant effect of latent sexting profile on the 8-sexting items of sending or receiving, consensual or non-consensual, with a partner or non-partner (*p* < .001). Follow-up post-hoc comparisons using the Games-Howell test indicated the frequency of the eight sexting experiences were statistically significant across latent profiles.

### Differences in Latent Sexting Profiles on Pressure to Sext

Four one-way ANOVAs were also used to examine whether the latent sexting profiles differed by perceived pressure to be involved in sexting (i.e., to send/receive sexts to/from a partner and non-partner). A corrected test, the Welch test was used for each one-way ANOVA given the unequal variances. There was a significant effect of latent sexting profile on the pressure to send “sexts” to a partner, *F*(2, 276) = 74.91, *p* < .001. Follow-up post-hoc comparisons using the Games-Howell test indicated that participants in the *high sexting* (*M* = 3.45, *SD* = 1.26) and *moderate sexting* (*M* = 2.97, *SD* = 1.34) profiles were significantly more likely to feel pressure to send “sexts” to their partner compared to participants in the *low sexting* profile (*M* = 1.54, *SD* = 1.03). There was no significant difference, however, between the *moderate* and *high sexting* profiles on the pressure to send “sexts” (*p* = .083). There was also a significant effect of latent sexting profile on the pressure to receive “sexts” from a partner, *F*(2, 224) = 75.68, *p* < .001. Games Howell post-hoc comparisons revealed that participants in the *high sexting* (*M* = 3.60, *SD* = 1.16) and *moderate sexting* (*M* = 2.77, *SD* = 1.32) profiles were significantly more likely to feel pressure to receive “sexts” from their partner compared to participants in the *low sexting* profile (*M* = 1.42, *SD* = 0.89). Participants in the *high sexting* profile were also significantly more likely to feel pressure to receive “sexts” from a partner than those in the *moderate sexting* profile (*p* < .001).

Similar differences were found for sending and receiving “sexts” to and from a non-partner. There was a significant main effect of latent sexting profile on the pressure to send “sexts” to a non-partner, *F*(2, 253) = 96.25, *p* < .001. Games Howell post-hoc comparisons revealed that participants in the *high sexting* (*M* = 3.58, *SD* = 1.21) and *moderate sexting* (*M* = 2.76, *SD* = 1.24) profiles were significantly more likely to feel pressure to send “sexts” to a non-partner compared to participants in the *low sexting* profile (*M* = 1.32, *SD* = 0.81). Further, participants in the *high sexting* profile were significantly more likely to feel pressure to send “sexts” to a non-partner than those in the *moderate sexting* profile (*p* < .001). Finally, there was a significant effect of latent sexting profile on the pressure to receive “sexts” from a non-partner, *F*(2, 206) = 36.83, *p* < .001. Games Howell post-hoc comparisons revealed that participants in the *high sexting* (*M* = 3.52, *SD* = 1.24) and *moderate sexting* (*M* = 2.69, *SD* = 1.33) profiles were significantly more likely to feel pressure to receive “sexts” from a non-partner compared to participants in the *low sexting* profile (*M* = 1.58, *SD* = 1.13). Participants in the *high sexting* profile were also significantly more likely to receive “sexts” from a non-partner than those in the *moderate sexting* profile (*p* < .001).

## Discussion

The goal of this study was to identify latent profiles of sexting behavior. Three latent profiles of sexting were found that were differentiated by the frequency of sending, receiving, and distributing sexts. Most adolescents reported little to no involvement in sexting behaviors. Those who were involved in moderate and high sexting profiles engaged in both consensual and non-consensual behaviors with a partner and non-partner and were more likely to experience problems with cyber bullying and victimization, with social media use, as well as problems regulating their behaviors and emotions. These findings are consistent with those of Mori et al. (2019), whereby youth that engaged in sexting more frequently were more likely to experience difficulties such as psychological distress and to report greater involvement in other risky behaviors. While engaging in no/low levels of sexting was not associated with harm, more frequent sexting that includes non-consensual as well as consensual sexting was associated with more problems. Thus, our findings support a normalcy approach which contends that consensual sexting is a normative risk-taking behavior and which recognizes non-consensual sexting as problematic and a type of sexual harassment (Henry et al., 2018; Jørgensen et al., 2019).

Contrary to our hypotheses, there were three latent profiles of sexting that represented the frequency of sexting rather than whether the sexting was consensual versus non-consensual or with a partner versus non-partner. The majority (81.4%) of the participants were in the *no/low sexting* profile, with 10.3% in the *moderate sexting* profile, and 8.3% in the *high sexting* profile. The latent profiles did not vary by gender or age. Findings indicated that the youth in the *no/low sexting* profile infrequently send and receive sexts and do not participate in non-consensual sexting or in distributing sexts. The youth in the *moderate sexting* profile primarily engage in sending and receiving consensual sexts with a partner and a non-partner and are less engaged in distributing sexts. Participants in the *high sexting* profile were more heavily involved in consensual and non-consensual sending and receiving as well as in distributing sexts, which may reflect a pattern of engaging in risky behaviors more generally (Mori et al., 2021). Although the profiles were differentiated based on frequency, there were some interesting differences among the profiles.

Adolescents in the *no/low sexting* profile do not sext or do so infrequently, with partners, and do not report feeling pressured to sext, and may represent adolescents who are at low risk. Sexting may represent a consensual healthy expression of intimacy and a way to explore sexual intimacy in their relationships. These youth reported greater self-regulation and engaged in less cyber bullying and reported less cyber victimization and less problematic social media use. The results are consistent with Mori et al. (2021) who suggest that youth who do not engage in sexting are at low risk for other risky behaviors and less likely to experience mental health problems.

In contrast, participants in the *moderate* and *high sexting* profiles represented one fifth of youth, who may engage in risky sexting and more generally, engage in more risky and problematic behavior. They reported less self-regulation, more cyber victimization, greater engagement in cyber bullying and more problematic social media use than those in the *no/low sexting* profile. Adolescents in the *moderate* and *high sexting* profiles might spend more time online and when online, engage in many different types of risky behavior. There appears to be a continuum of risk, with youth in the *high sexting* profile reporting the highest level of involvement in both cyber bullying and victimization. It may be important to understand this constellation and the developmental pathway of these risky behaviors, for example, whether dysregulation leads to high sexting frequency. Longitudinal research is required to untangle such critical associations.

The findings of the current study are supported by a meta-analysis of the association among sexting, sexual behaviors, and mental health, which found that youth that sexted were more likely to experience mental health risks than those that were not engaged in sexting (Mori et al., 2019). A more recent study showed that non-consensual sexting was associated with poor psychosocial outcomes among early adolescents (Lu et al., 2021). Mori et al.’s (2021) latent class analysis on sexting and sexual behaviors amongst youth found that a quarter of the sample had a higher probability of sexting and sexual activity, and higher probabilities of sexually risky behavior, with the study concluding that risky behaviors may cluster together.

This continuum of risk was also reflected in the results related to the pressure to sext. Our results indicate that participants in the *moderate* and *high sexting* profiles were more likely to report feeling pressured to send and receive sexts, both with a partner and non-partner than were participants in the *no/low sexting* profile. Those in the *moderate* and *no/low sexting* profiles were less likely to feel pressure to send “sexts” to a non-partner than those in the *high sexting* profile and to report non-consensual distribution. With respect to receiving sexts, participants in the *moderate sexting* profile were less likely to feel pressure than those in the *high sexting* profile.

Pressure is associated with an increased frequency of sexting. The pressure to send and receive sexts in the *moderate and high sexting* profiles is problematic and may represent “unwanted but consensual” sexting (Drouin & Tobin, 2014). The reported pressure might reflect that consent is complicated. Both girls and boys may report experiencing pressure, although there may be different motivations behind the pressure. Girls may feel pressure to send and receive sexts, for such reasons as a boy threatening to break up with them or label them pejoratively if they do not send sexts (Mishna et al., 2023). Boys may experience pressure from other boys to obtain sexts from girls due to gender norms (e.g., gain status; J. Ringrose et al., 2021). Research is required to understand the nature of the pressure, what the pressure represents, and how it is experienced within the context of adolescent relationships.

### Limitations

First, adolescent experiences of sexting were only measured at one assessment. Although the findings provide a snapshot of the profiles for adolescent sexting experiences, a longitudinal study is needed to determine how adolescents may transition across latent sexting profiles over time. Second, the quantitative focus was a strength of the study however, a mixed-methods study that may include focus groups or interviews would allow for greater insight into what is considered consensual or non-consensual sexting as well as what pressure to sext may look like for adolescents. Third, internal reliabilities could not be determined for measures of cyber bullying and victimization, and problematic social media use due to individual items or dichotomous response options. Both types of assessments are considered appropriate within the literature (e.g., Cosma et al., 2020) but including a more comprehensive measure may help to determine if particular aspects of cyber bullying, cyber victimization, or problematic social media use are more strongly associated with particular latent sexting profiles. Finally, adolescents’ experiences with technology were only assessed via self-report. As a result, it is possible that some online behaviors such as sexting or cyber bullying were underreported (Mori et al., 2021). Given that sexting and cyber bullying may occur privately within the context of two adolescents, including assessments with multiple informants such as peers or parents may not yield reliable information. It is also possible that some parents were not comfortable allowing their child to participate in the research due to the potentially sensitive nature of the questions. Thus, we cannot ensure the sample was representative of Canadian adolescents as intended.

### Implications and Conclusion

The study findings suggest that most adolescents reported limited to no involvement in sexting and that their experiences were primarily with their partner. The conditions and frequency with which about one-fifth of youth send, receive, and distribute sexts, however, was associated with increased harm and with other risky online behaviors. An important finding is the association between experiencing pressure to sext and a greater likelihood to engage in other risky behaviors. There are several key implications of these findings. Mental health practitioners are well situated to offer support to parents and educators as well as youth in recognizing factors, such as consent, gender, and social pressure, that influence youth’s experiences with sexting (Mishna et al., 2020, 2023). This implication is consistent with responding through a normalcy perspective to youth sexting, which recognizes both the benefits and risks of sexting that is consensual as well as the troublesome phenomenon of non-consensual image sharing. Social supports are essential in reducing the harms of normative risk-taking sexual behaviors, such as distinguishing consensual and non-consensual sexting by delimiting the ways in which non-consensual sexting constitutes a form of online sexual harassment. In conclusion, our findings support the normalcy approach to education with youth, parents, and teachers, which considers some sexting among healthy developmental behaviors.
